# ILRC: a hybrid biomarker discovery algorithm based on improved L1 regularization and clustering in microarray data

**DOI:** 10.1186/s12859-021-04443-7

**Published:** 2021-10-22

**Authors:** Kun Yu, Weidong Xie, Linjie Wang, Wei Li

**Affiliations:** 1 College of Medicine and Biological Information Engineering, Northeastern University, Shenyang, China; 2 School of Computer Science and Engineering, Northeastern University, Shenyang, China; 3 Key Laboratory of Intelligent Computing in Medical Image (MIIC), Northeastern University, Ministry of Education, Shenyang, China

**Keywords:** Microarray, Feature selection, Biomarker, Machine learning

## Abstract

**Background:**

Finding significant genes or proteins from gene chip data for disease diagnosis and drug development is an important task. However, the challenge comes from the curse of the data dimension. It is of great significance to use machine learning methods to find important features from the data and build an accurate classification model.

**Results:**

The proposed method has proved superior to the published advanced hybrid feature selection method and traditional feature selection method on different public microarray data sets. In addition, the biomarkers selected using our method show a match to those provided by the cooperative hospital in a set of clinical cleft lip and palate data.

**Method:**

In this paper, a feature selection algorithm ILRC based on clustering and improved L1 regularization is proposed. The features are firstly clustered, and the redundant features in the sub-clusters are deleted. Then all the remaining features are iteratively evaluated using ILR. The final result is given according to the cumulative weight reordering.

**Conclusion:**

The proposed method can effectively remove redundant features. The algorithm’s output has high stability and classification accuracy, which can potentially select potential biomarkers.

## Background

Microarray data is a valuable tool for analyzing gene expression profiles [1]. This kind of data usually contains a small number of biological or clinical samples and a large number of genes (features) that are not related to the target disease [2]. In addition, microarray data shows a high complexity, i.e., genes are direct- or inter-related, which results in a high degree of redundancy. These features make many machine learning algorithms incompetent to microarray data with low robustness and poor classification accuracy [3]. Therefore, it is essential to find a suitable method to reduce the number of features for constructing a model, to improve the classification accuracy and robustness.

Extensive research has shown that feature selection is crucial for building statistical models when mining large datasets of high dimension, especially for those generated from microarray and mass spectra analysis [4]. It is a significant step forward for selecting biomarkers in biological data. Standard feature selection methods can be divided into filter methods, wrapper methods, and embedded methods [5]. Some advanced hybrid feature selection methods [6–12] have been reported, which can achieve a higher classification accuracy with a smaller number of features using public gene datasets. These methods are described in detail in the Related work section.

Meanwhile, results from the proteomic analysis are experiencing the same issue as high-dimensional gene expression data based on advanced mass spectroscopy. Many of identified proteins might be irrelevant to the disease and have high correlations among each other. Machine learning methods have shown a significant advantage in dealing with this genetic and clinical data in the past few years [13–15]. Recently, these methods have been optimized to process proteomic/metabolic data acquired from mass-spectrometry in some diseases [16–18].

Existing researches have dedicated to improving the accuracy of prediction models with a smaller number of features. However, for the discovery of markers, the reliability and stability of the results must be emphasized. If part of the data is updated or modified, and the markers selected by the algorithm change significantly after the disturbance of the data set, ,which makes the original ones are no longer reliable for researchers [19]. Although literature [20] emphasizes the importance of feature stability and designs a sorting algorithm, this method does not consider result stability in the feature selection process simultaneously.

Meanwhile, there is little literature explaining and validating the clinical meaning of the selected genes or proteins in these literatures. Despite the high classification accuracy, a priori knowledge or manual verification is necessary for assessing the biological plausibility and the specificity of any biomarker clusters identified by the algorithm. Taken alpha-fetoprotein (AFP) as an example, it is a well-known biomarker for a variety of problems of pregnancy, such as open neural tube defects, abdominal wall defects and Down’s syndrome [21]. However, the level of AFP is also elevated in omphalocele, gastroschisis, and sacrococcygeal teratoma, etc. [22], which makes it insufficient to distinguish the actual fetal abnormalities.

**Fig. 1:**
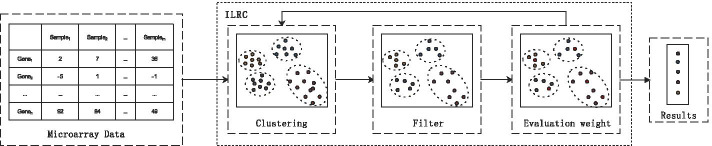
Proposed method framework. This method first clusters the features, and filters the features that are highly correlated with the node with the highest weight in each sub-cluster, and then uses the improved L1 regularization method to evaluate the weight of each feature for multiple iterations. Finally, the output feature subsets are sorted according to feature weights

In this paper, a hybrid method combining improved L1 regularization and cluster-based (ILRC) biomarker selection is proposed to solve the above problems. The overall framework is shown in Fig. 1. The method firstly clusters the features and filters the part of the features that has the highest correlation with the seed node in each sub-cluster. Then it uses the improved L1 regularization method to evaluate the weight of each feature for multiple iterations, and finally it sorts the output features according to the weight of the feature subset. The results on the public data set and a set of cleft lip and palate disease proteomics data (provided by the cooperative hospital, biomarkers have been verified) prove the effectiveness of this method.

The contribution of this article is reflected in the following aspects:

1. A hybrid feature selection method is designed for biomarker selection. This method focuses on the stability and validity of the results during the feature selection process while ensuring a high accuracy rate.
2. A combination of clustering algorithm with filter method and embedded method for biomarker selection.
3. Comparatively evaluation of the performance of various classifiers on microarray data.
4. Introduce a data set that can be used for biomarker verification to prove the validity of the results.

### Related work

Traditional feature selection methods can be divided into filter, wrapper and embedded methods. In general, the filter method does not involve classification models. It only uses the intrinsic characteristics of the data to measure the important feature score [23]. Comparing to other feature selection methods, it has a lower time complexity, which allows a flexible arrangement to combine with other feature selection algorithms for data preprocessing achieving, noise removal and dimensional reduction [24–26]. The common filter methods mainly contains ReliefF [27], T-Test [28], Chi-squared test [29], and maximal information coefficient (MIC) [30], Gini Index [31], Kullback–Leibler divergence [32], Fisher score [33], Laplace operator score [34].

Wrapper methods usually add a classifier that involves evaluating the subset of features [35]. It takes classifiers as an integral part of the algorithm and evaluates the importance of selected features according to the classifier performance, which usually generates a better model accuracy. Common wrapper methods incorporates stability selection [36], recursive feature elimination (RFE) [37], genetic algorithm (GA) [38], K-nearest neighbor (KNN) [39] and particle swarm optimization (PSO) [40].

The idea of embedded methods is similar to wrapper methods. They both involve classifiers. However, in the embedded method, the selection of feature subsets is directly embedded in the classifiers. In other words, the feature selection is carried out simultaneously with the training of the classifier. The common embedded method comprises supported vector machine recursive feature elimination (SVM-RFE), decision tree (DT) [41], random forest algorithm (RF) [42], and Lasso regression (LR) [43].

However, recent studies have shown that hybrid feature selection methods can simultaneously take the efficiency advantages of filter method and the accuracy advantages of warpper method to achieve superior performance [44]. In addition, some studies have also figured out the data imbalance problem commonly appeared in microarray datasets [45, 46].

For the microarray data of DNA, Lu et al. combined mutual information maximization (MIM) and adaptive genetic algorithm (AGA) to propose a hybrid feature selection algorithm, MIMAGA-Selection [6]. The method effectively reduces the dimensionality of the original gene expression dataset and achieves the goal of eliminating data redundancy. It uses MIM to find genes in the same category that are highly dependent on other genes. Experimental results show that the accuracy of the MIMAGA-Selection method outperforms the three existing algorithms selected, ReliefF, sequential forward selection (SFS) and MIM. To verify the validity of the genes selected by the MIMAGA-Selection method, the paper of MIMAGA-Selection includes the backpropagation neural network (BP), the support vector machine (SVM), extreme learning machine (ELM) and the regularized extreme learning machine (RELM).

Salem et al. proposed a new method for finding biomarkers from gene microarray data that combines information gain (IG) and a standard genetic algorithm (SGA) for feature selection, called IG/SGA [7]. The information gain is used for the initial feature selection to reduce redundant features and is used to improve the efficiency of the genetic algorithm. The genetic algorithm is then used for further feature selection, and finally, genetic programming (GP) is used to build the final classification model. Experimental results on the seven datasets employed show that, compared to other hybrid feature selection methods, the method proposed in this paper generally achieves the best classification accuracy and achieves 100% classification accuracy on two datasets.

Alshamlan et al. proposed a new feature selection algorithm, called the minimum redundancy maximum relevance (mRMR) method, and combined it with an Artificial Bee Colony algorithm (ABC) to filter biomarkers from gene microarray data [8]. mRMR is used as a filtering method to reduce the number of features and improve the efficiency of the ABC algorithm. The method uses a support vector machine (SVM) as a classifier and compares with mRMR combined with genetic algorithm (mRMR-GA) and mRMR combined with particle swarm optimization algorithm (mRMR-PSO) on five datasets. The results show that the mRMR-ABC method is able to provide a higher classification accuracy when using a small number of features.

Jain et al. combine correlation-based feature selection (CFS) with an improved binary particle swarm optimization (iBPOS) to propose a two-stage hybrid feature selection method for biomarker selection of microarray data [9]. Like other advanced hybrid feature selection methods, CFS is used to improve the particle swarm algorithm’s performance. The method uses a Bayesian model as a classifier and experiments have been conducted on 11 different microarray datasets. Results comparing the method with some advanced feature selection methods show that the method generally outperforms the comparison algorithm in terms of classification accuracy and achieves 100% classification accuracy on seven datasets.

Moradi et al. proposed a hybrid feature selection method based on particle swarm optimization (PSO) algorithm [10]. The main idea of the method is to sneak in a local search strategy to guide the search and selection process of the particle swarm optimization algorithm. A comparison with five advanced feature selection methods on 13 datasets was carried out. The results showed that the proposed method could improve the classification accuracy and had significant advantages over other methods.

Shreem et al. proposed a two-stage feature selection hybrid method for solving the biomarker identification problem for microarray data [11]. The method combined symmetric uncertainty (SU) and harmonious search algorithm (HSA), abbreviated as SU-HAS. In the first step, the SU method is used to remove redundant features, while the second stage used HAS to select the best feature genes. In the experiments with 10 microarray data, the method obtained the highest classification accuracy in five datasets compared to other advanced feature selection methods.

Two hybrid feature selection methods based on the fast correlation based filter (FCBF) algorithm were studied by Djellali et al. [12]. The first method was based on a genetic algorithm (FCBF-GA) and the second method was based on a particle swarm optimization algorithm (FCBF-PSO). The first stage of the method used FCBF for feature filtering and then the results were fed to either the genetic or particle swarm algorithm. The method was evaluated on four microarray datasets using a support vector machine as a classifier, and the results showed that FCBF-PSO outperformed FCBF-GA.

## Results

In this section, we show the experimental results according to the processing flow of the feature engineering pipeline. In the data preprocessing process, the impact of different sampling methods on the unbalanced data set is compared. In the process of feature selection, we compared our proposed method with typical feature selection methods. In the model-building stage, we compared the effectiveness of different classification models. Finally, in the result evaluation, the proposed method was compared with the advanced feature selection method. In addition, we also evaluated the proposed method on the cleft lip and palate (CLP) dataset.

### Sampling results of unbalanced data sets

Table 1 shows the evaluation of the results after sampling on five imbalanced data sets using two different sampling methods (oversampling and combined sampling). To ensure the reliability of the results, the experiment selected multiple classification models and selected the same number feature. These methods included support vector machine (SVM), Gaussian Bayes (GB), decision tree (DT), neural network (NN) and K-nearest neighbor (KNN). These classifiers are commonly used in bioinformatics analysis, and have been proven to have good performance [47–49]. It can be see that combined sampling achieves the best results.

**Table 1:** Classification accuracy of different sampling methods on unbalanced datasets

	Datasets	SVM	GB	DT	NN	KNN
Origin datasets	ALL1	1.0000	1.0000	0.9843	1.0000	1.0000
	ALL3	0.8080	0.8640	0.7680	0.8240	0.8080
	ALL4	0.9567	0.9053	0.8053	0.9129	0.8930
	DLBCL	0.9875	0.9750	0.8558	0.9875	0.9750
	Myeloma	0.9018	0.8965	0.7114	0.8842	0.8382
Over sampling	ALL1	1.0000	1.0000	1.0000	1.0000	1.0000
	ALL3	0.8261	0.8271	0.8371	0.9307	0.7326
	ALL4	0.9704	0.9254	0.8644	1.0000	0.8729
	DLBCL	0.9482	0.9312	0.9141	0.9739	0.8790
	Myeloma	0.8793	0.8613	0.8359	0.8687	0.8100
Combined sampling	ALL1	1.0000	1.0000	1.0000	1.0000	1.0000
	ALL3	0.8766	0.7926	0.7918	0.9456	0.7616
	ALL4	0.9704	0.9259	0.7610	0.9630	0.8667
	DLBCL	0.9913	0.9913	0.8877	1.0000	0.9830
	Myeloma	0.8943	0.8650	0.8250	0.8725	0.8100

### Comparison results of feature selection methods

**Fig. 2:**
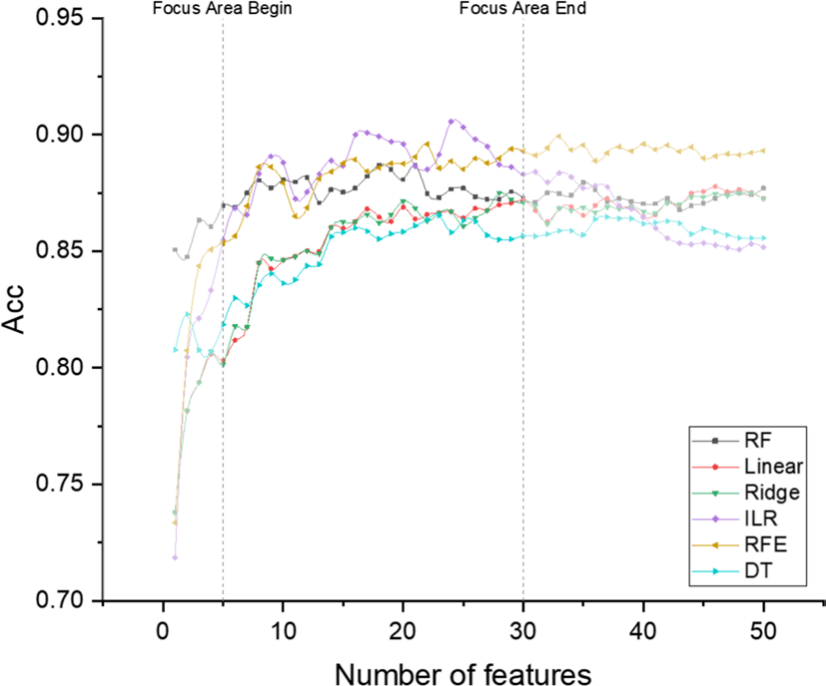
Classification accuracy of different classification models. The result is the average of all data sets. The horizontal axis represents the number of features selected, and the vertical axis represents the average classification accuracy. The Focus Area in the figure is the range of the number of features that researchers are concerned about

Figure 2 shows the classification accuracy of different classification models on the balanced datasets. These methods are random forest (RF), linear regression model (Linear), Ridge regression model (Ridge), improved L1 regularization based linear regression model (ILR), recursive feature elimination (RFE), and decision tree (DT).

Usually, in microarray data analysis, the number of features that researchers pay attention to is between 5 and 30, which is also the focus of our attention. It can be seen that ILR has achieved better results in this area, and ILR is also part of the hybrid algorithm we proposed.

**Fig. 3:**
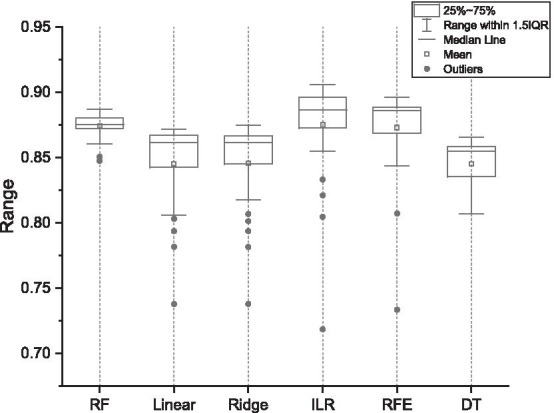
Classification accuracy of different feature selection mehtods. The box graph represents the classification accuracy of cross validation, the horizontal axis represents the feature selection method adopted, and the vertical axis represents the classification accuracy

**Fig. 4:**
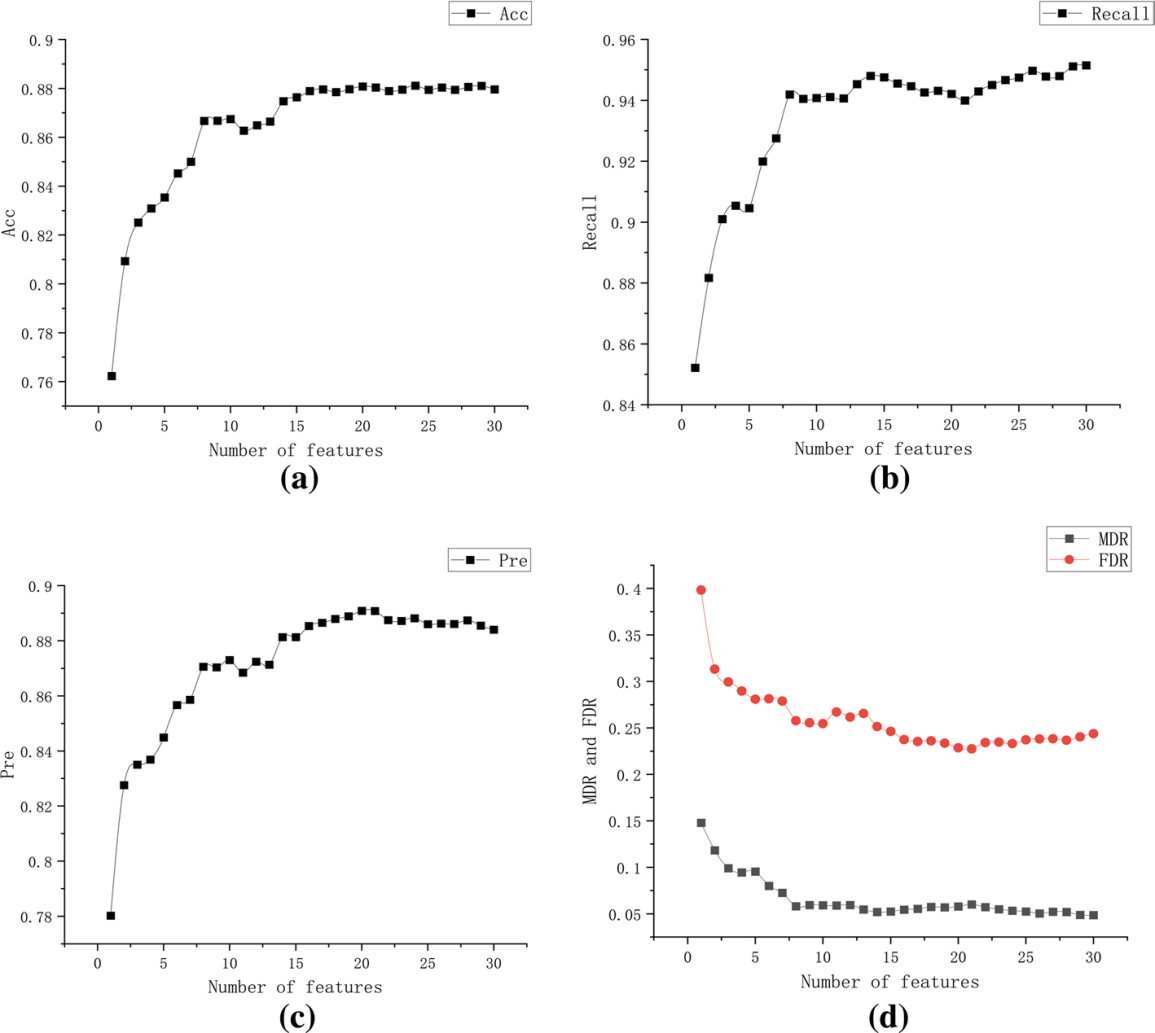
Summary results for each evaluation indicator under different number of features. **a** The horizontal axis indicates the number of features, and the vertical axis indicates the classification accuracy rate. **b** The horizontal axis indicates the number of features, and the vertical axis indicates the recall rate. **c** The horizontal axis indicates the number of features, and the vertical axis indicates the precision rate. **d** The horizontal axis represents the number of features, and the vertical axis represents false discovery rate (FDR) and miss discovery rate (MDR) for different number of features

Figure 3 shows the average classification accuracy of different methods when the number of features is limited to 30. Figure 4 shows the changes of different indicators. The results indicate that in the area of interest, the evaluation indicators can reach a stable state.

### Effect evaluation of different classification models

**Fig. 5:**
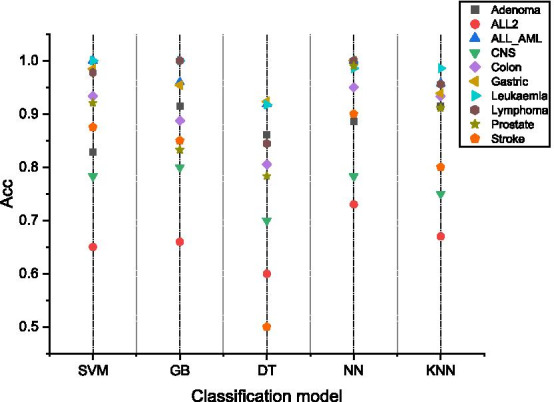
Classification accuracy of different classification models. The horizontal axis represents the classifier, the vertical axis represents the classification accuracy rate, and each point represents a dataset. The result in the figure is the average classification accuracy on all data sets

Figure 5 shows the average classification accuracy of different classification models on different data sets. SVM, GB and NN can provide good classification accuracy and are suitable for processing microarray data. In this article, we use SVM as the classifier for all experiments.

### Evaluation of the effectiveness of the proposed method ILRC

Table 2 shows the comparison between the proposed method and the advanced hybrid feature selection algorithm on the public microarray dataset. The number of features and classification accuracy are evaluated respectively. It can be seen that ILRC can achieve higher classification accuracy with a smaller number of features.

**Table 2:** Comparison on the dataset Colon, Lymphoma and Leukemialy with 7 advanced hybrid feature selection algorithms

Dataset	Colon		Lymphoma		Leukemial	
	Acc	Features	Acc	Features	Acc	Features
Lu et al. [6]	83.41	202	/	/	96.67	44
Salem et al. [7]	85.48	60	/	/	97.06	3
Alshamlan et al. [8]	96.77	15	100	5	100	14
Jain et al. [9]	/	/	100	24	100	4
Moradi et al. [10]	/	/	50	87.71	89.28	100
Shreem et al. [11]	87.53	9	100	10	100	26
Djellali et al. [12]	96.3	1000	/	/	/	/
ILRC	95	5	100	9	100	5

### Effect evaluation of ILRC on CLP data set

In order to verify the performance of the proposed feature selection method in real data sets, we tracked the ranking of three known biomarkers in the algorithm in the CLP data set. These biomarkers have been labeled and verified to be effective.

To ensure the stability of the results, we repeated 1042 experiments (consistent with the number of features) in different methods and respectively took the average weight ranking and average frequency corresponding to each feature as the final ranking. The feature weight comes from the classifier and is generated in the model building. The average frequency is the average number of occurrences of the feature in repeated experiments.

Table 3 shows the ranking of three biomarkers in different methods. In the experiment, if the threshold is set too small, redundant features cannot be filtered. On the other hand, if the threshold is set too large, some important features will be filtered out. Usually a threshold of 30% can achieve the best effect, and 20–35% is the recommended threshold interval. ILR is an improved L1 regularization method. ILRT and ILRM represent ILR methods that use T-test and mutual information as a preprocessing method, RF represents random forest, and DT represents Decision tree. For ILRC, we set different weights, and the weight coefficient corresponds to the *p*% node with the highest correlation with the seed node in the method. It can be seen that the three known biomarkers rank better than other methods in ILRC.

**Table 3:** Ranking of characteristic proteins on CLP datasets by different methods, “/” indicates that the corresponding feature is within the number of cycles, and the feature has not been selected by the algorithm once

	ID	ILR	ILRT	ILRM	RF	DT	ILRC (20%)	ILRC (25%)	ILRC (30%)	ILRC (35%)
Count	P31946	22	56	46	102	158	**7**	10	20	12
	P35968	87	/	/	83	85	**14**	29	18	19
	P62258	55	48	/	14	52	13	16	**12**	76
Weight	P31946	61	69	61	96	158	21	18	20	**15**
	P35968	68	/	/	139	88	27	27	**15**	20
	P62258	39	13	/	97	57	9	15	**1**	31

Bold indicates that the protein ranked best at the corresponding threshold

## Discussion

From the results in Table 1, it can be seen that sampling for unbalanced data sets is necessary for the data preprocessing stage. The effects of oversampling and combined sampling methods on classification accuracy were tested [50]. Combined sampling can significantly improve the classification accuracy under different classification models.

The average classification accuracies of different feature selection methods with multiple datasets were compared in Figs. 2 and 3, where ILR is part of our proposed hybrid approach. It can be seen that ILR achieves high results within the interval of the number of features of interest to the researchers. Figure 4 evaluates the different metrics for all results within the number of features we focus on. The results show that the classifier can achieve the highest evaluation within the number of features we focus on, while the inclusion of more features decreases the model performance. This is because some irrelevant and redundant features are fed into the classifier, and our proposed approach will focus on removing such features.

In Fig. 5, the classification performance of different classification models was evaluated for microarray data, and for each classifier, we input 15 features selected by the proposed method. The results show that SVM, GB and NN are more suitable for the classification task with microarray data. However, the results differ slightly on different datasets. To solve this problem, pre-experiments may be needed to select classifiers suitable for microarray datasets.

In Table 2, our proposed method was compared with published advanced hybrid feature selection methods. ILRC performs very stably, achieving over 95% accuracy for less than ten features and 100% accuracy on the Lymphoma and Leukemia datasets. Although some of the results lagged slightly behind the other methods, these differences were not significant. Overall, we have reason to believe that ILRC can achieve excellent performance on most datasets.

Table 3 evaluates the effect of ILRC on a CLP dataset in which a subset of biomarkers have been labeled and validated by clinical experts. P31946, P35968 and P62258 are proteins that have been validated as clinical biomarkers, and it can be seen that ILRC is better than some traditional feature selection methods and ILR algorithms based on different combinations of filtering methods for the discovery of such proteins. The results indicate that ILRC results are stable and have the potential to discover biomarkers of clinical significance.

ILRC combines the clustering algorithm with the hybrid feature selection algorithm (T-Test and ILR), which can effectively filter redundant features, select biomarkers with diagnostic significance, and ensure high classification accuracy. However, the ILRC algorithm does not involve ensemble learning. Usually for feature selection tasks, especially the selection of biomarkers, the use of ensemble models and feature ranking fusion technology can improve the robustness of the results. We will focus on this direction in future research.

## Conclusion

Microarray data analyzes the genetic differences of tissues and cells. In clinical medicine, effective gene selection can greatly enhance the process of disease prediction and diagnosis. Genes useful for predicting cancer types may also provide support for the pathogenesis and pharmacology of cancer [51]. The ILRC method proposed in this paper clusters the features and establishes seed nodes in the sub-clusters according to the evaluation rules. It deletes the features with higher redundancy of the seed nodes, and uses the improved L1 regularization method to evaluate the remaining feature subsets before the best subset are finally chosen. Multiple comparison experiments and verification experiments on real data sets have proved the accuracy and stability of ILRC, and it has the potential to discover clinically significant biomarkers.

In our research, we found that the feature subsets generated by different models may have large deviations. Researchers need a stable subset of features. Supposing part of the data is updated or modified, the markers selected by the algorithm change significantly after the disturbance of the data set. These markers may be unreliable for researchers [19]. The following research will focus on the stability of features and the multi-model feature ranking fusion method. Related methods have been reported in the literature [20], and excellent results have been achieved. In addition, in future work, we will evaluate the effects of related reports on the CLP dataset.

## Method

### Datasets and evaluation index

There are 17 different datasets used in this paper, including 17 publicly available genetic datasets and CLP protein dataset, as shown in Table 4. The 16 publicly available datasets include diffuse large B-cell lymphoma (DLBCL) [52], Prostate [53], acute lymphoblastic leukemia (ALL; subdivided into four subtypes based on different phenotypes) [54], central nervous system embryonal tumor (CNS) [55], Lymphoma (Lym) [56], Adenoma [57], Colon [58], Leukaemia [59], Myeloma [60], Gastric [61], Stroke [62] and Cleft lip and palate (CLP). Among them, DLBCL, Colon, Leukaemia, Myeloma, ALL1-4, and CNS datasets are imbalanced.

**Table 4:** The data set involved in this article, Ratio represents the unbalanced ratio of the data set

ID	Dataset	Pos/Neg	Samples	Features	Ratio	Summary
1	Adenoma	18/18	36	7457	1.00	Colon adenocarcinoma (18) and normal (18)
2	ALL_AML	47/25	72	7129	1.88	ALL (47) and AML(25)
3	ALL1	95/33	128	12,625	2.88	B-cell (95) and T-cell (33)
4	ALL2	65/35	100	12,625	1.86	Patients that did (65) and did not (35) relapse
5	ALL3	24/101	125	12,625	0.24	With (24) and without (101) multidrug resistance
6	ALL4	26/67	93	12,625	0.39	With (26) and without (67) the t(9;22) chromosome translocation
7	CNS	39/21	60	7129	1.86	Medulloblastoma survivors (39) and treatment failures (21)
8	Colon	40/22	62	2000	1.82	Tumour (40) and normal (22)
9	DLBCL	58/19	77	7129	3.05	DLBCL patients (58) and follicular lymphoma (19)
10	Gastric	29/36	65	22,645	0.81	Tumors (29) and non-malignants (36)
11	Leukaemia	47/25	72	7129	1.88	ALL (47) and AML (25)
12	Lymphoma	22/23	45	4026	0.96	Germinalcentre (22) and activated B-like DLBCL (23)
13	Myeloma	137/36	173	12,625	3.81	Presence (137) and absence (36) of focallesions of bone
14	Prostate	52/50	102	12,625	1.04	Prostate (52) and non-prostate (50)
15	Stroke	20/20	40	54,675	1.00	Ischemic stroke (20) and control (20)
16	CLP-prot	30/30	60	1042	1.00	Mothers with CLP-affected fetuses(30) and with normal controls (30)

Cleft lip and palate (CLP) are common congenital orofacial defects of a baby’s lip or mouth. It is originated from a failure joint of tissue and special cells during the formations of lip and mouth (palate), which happens between the fourth and ninth weeks of pregnancy [63]. Babies with cleft lip and/or palate have an opening in their upper lips (and/or the front part of their palates). In some severe cases, this cleft goes even further to babies’ noses. There are approximately 1 out of 700 live births affected by orofacial clefts on average [64]. Children with these defects suffer from difficulties in feeding, speaking and sometimes hearing problems caused by infections.

In general, this congenital malformation can be divided into two types: syndromic which is mainly caused by genetic mutations, and non-syndromic with more complicated inducing factors including a combination of genes and environment, such as smoking, diabetes, the usage of certain medicines before and during pregnancy [65–68]. Over hundreds of genes have been mapped to the genetic causes of these defects indicating a strong genetic component to the disease development [69]. However, the underlying mechanism is still unclear.

Routine prenatal diagnosis method of CLP is conducted by anatomic ultrasonography between 18 and 20 weeks’ gestation [70]. Repairing surgeries can be performed directly after babies are born and within the first 18 months of life [71]. Apart from the fetal position and physical conditions of the mother, detection sensitivity of any abnormalities in an ultrasound screen is highly dependent on the instruments used and the experience of medical sonographers. In most cases, further genetic or biochemical tests are carried out to gain a definitive diagnosis. The use of biomarkers provides great promise for aiding clinical diagnosis with the potential to detect early signs of fetal abnormalities. Early diagnosis of such defects will allow doctors to have a comprehensive treatment plan.

In this paper, the CLP dataset is the only dataset in which biomarkers have been tagged and validated by clinical doctors. The dataset contains 60 maternal serum samples (30 mothers with CLP-affected fetuses and 30 health babies as normal controls) that are collected between gestational weeks 22–30 for this study. 1042 proteins are identified by an iTraq based proteomics analysis. The serum samples were collected from pregnant women who underwent prenatal examination in a partner hospital. During the mass spectrum analysis experiment, every ten blood samples were mixed into one sample, so the actual sample size of the dataset is only six.

In this paper, the Accuracy, Precision, and Recall Rate, which are widely used in existing studies, are used as the primary evaluation metrics. We also introduce False Discovery Rate (FDR) and Miss Discovery Rate (MDR) to evaluate the selected features and classification models. The estimation of the fdr and mdr are requirements for the analysis and documentation of mass spectrometry data according to the Paris guidelines of Molecular and Cellular Proteomics [72]. These evaluation indicators are defined in the following formulas, where *P* and *N* represent the number of positive and negative samples, *TP* and *TN* represent the number of positive and negative samples predicted correctly, *FP* and *FN* represent the number of positive and negative samples predicted incorrectly. The evaluation indicators used in this paper are calculated as Eq. (1).

1 $$\begin{aligned} \begin{aligned} FDR&=\frac{FP}{TN+FP} \quad Accuracy=\frac{TP+TN}{P+N}\\ MDR&=\frac{FN}{TP+FN }\quad Precision=\frac{TP}{TP+FP}\\ \end{aligned} \end{aligned}$$

In this experimental section, a tenfold cross-validation method is used and averaged as the final classification accuracy to ensure consistency with the experimental approach of the comparison articles. For datasets with a smaller number of samples, the “leave-one-out” method is used to validate and average these data so that the result is closest to the expected value in the entire training and test set.

### Improved L1 regularization method

The Improved L1 Regularization method, also known as stability selection, is based on a combination of sampling and feature selection [73]. This method is a complement to the L1 regularization method: when the L1 regularization method is confronted with a set of associated features, it tends to select only one of the features. Stability selection uses randomization techniques. The main idea is to run the feature selection algorithm on different subsets of data and subsets of features, constantly repeating and eventually aggregating the feature selection results. Ideally, important features will score close to 1, less important features will score somewhere between 0 and 1, and the most useless features will score close to 0.

An important feature of the L1 regularization method is the ability to generate sparse matrices of feature weight coefficients, i.e., the coefficients of some features become zero, so that feature selection can be achieved based on feature weights. Therefore L1 regularization is often used for feature selection of high dimensional data. However, to obtain the correct results, the L1 regularization method requires the data space to meet specific conditions. In addition, if there is a high correlation between features, the L1 regularization method is prone to distortion, making it difficult to achieve a high classification accuracy. The L1 regularization method is also very sensitive to the regular term coefficient alpha, so it is critical to choose the right parameters. When facing data with high dimensional and small sample, the number of features selected by L1 regularization method is less than $\min (n, p)$, which leads to less stable and reproducible parameters obtained by estimation [74].

The improved L1 regularization algorithm’s core idea is first random sampling, and then use a feature selection model to evaluate the selection [75]. When the feature correspondence coefficient is non-zero, the feature is considered to be selected. The algorithm repeats the above process several times to get the frequency of each selected feature, and the feature with the higher frequency is selected as the final selection result. According to the improved L1 regularization framework, it is easy to see that it allows the selection of appropriate methods according to the sample space, which also makes the stability selection framework has a broader application scenario. At the same time, stability selection weakens the sensitivity of the final results to the regularization coefficient $\alpha$, which greatly reduces the workload; stability selection is able to effectively control false positives, especially on high-dimensional small sample data, and this advantage is more obvious.

### K-means clustering method

Removing irrelevant features does not negatively affect the clustering accuracy and can reduce the required storage and computation time from a clustering perspective. Therefore, clustering algorithms are often used as one of the pre-processing methods to remove redundant features before feature selection [76]. For the sample set $D=\left\{ x_{1}, x_{2}, x_{3}, \ldots , x_{m}\right\}$, the K-means algorithm is the least square error *E* for the clustering partition $C=\left\{ C_{1}, C_{2}, \ldots , C_{k}\right\}$ as shown in Eq. (2).

2 $$\begin{aligned} E=\sum _{i=1}^{k} \sum _{x \in C_{i}}\left\| x-\mu _{i}\right\| _{2}^{2} \end{aligned}$$

where $u_{i}=\frac{1}{\left| C_{i}\right| } \sum _{x \in C_{i}} x$ is a vector of mean values of $C_i$.

Equation (2) depicts the degree of closeness of the intra-cluster samples around the cluster mean vector, with the smaller the *E* value, the greater the similarity of the intra-cluster samples.

### Improved L1 regularization clustering based feature selection method (ILRC)

A hybrid feature selection method, improved L1 regularized clustering-based (ILRC) for biomarker discovery is proposed in this subsection. This method uses T-test and improved L1 regularization (ILR) method as a hybrid feature selection method, and combines the improved K-Means clustering algorithm into ILR [77]. The experimental results show that the proposed method can obtain higher classification accuracy than most traditional feature selection methods and some advanced hybrid algorithms. Moreover, the CLP dataset experiments show that the addition of K-means can effectively improve the clinical interpretability of the selected features.

**Fig. 6:**
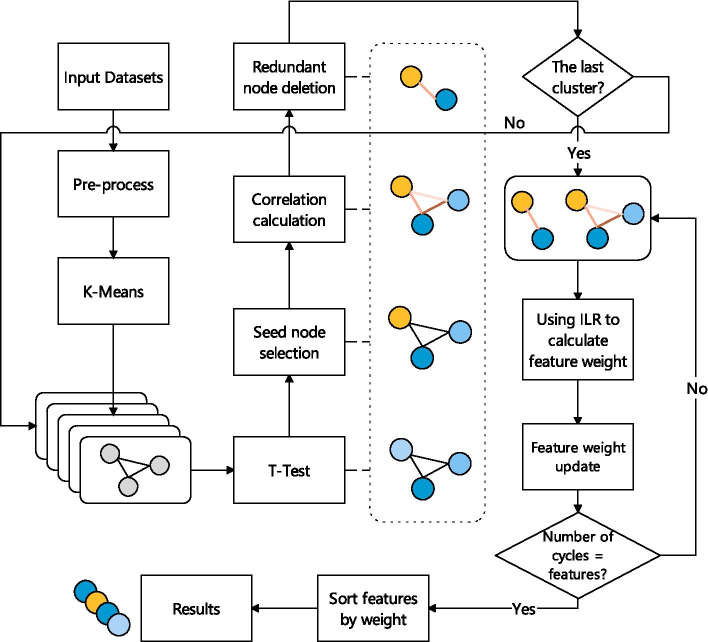
ILRC feature selection process. After the input data is preprocessed, the clustering operation is performed. After clustering, the features are sorted by T-test, and the seed node is selected. Then the correlation between the remaining nodes and the seed node is evaluated, and redundant features are deleted. Finally, use ILR to assess the remaining features’ weight, repeat the experiment, and output the results according to the order of features

The overall flow of the algorithm is shown in the Fig. 6. In ILRC, the data is firstly clustered using the K-means, the number of clusters (*k*) is determined using the elbow method [78]. The original dataset is divided into *k* sub-datasets (clusters), so each cluster’s features are similar. For each cluster, the *p* value of each feature (node) is calculated using the T-Test method. For each feature $x_i$, its *p* value is calculated as Eq. (3).

3 $$\begin{aligned} P=\frac{{\bar{X}}_{1}-{\bar{X}}_{2}}{\sqrt{\frac{\left( n_{1}-1\right) S_{1}^{2}+\left( n_{2}-1\right) S_{2}^{2}}{n_{1}+n_{2}-2}\left( \frac{1}{n_{1}}+\frac{1}{n_{2}}\right) }} \end{aligned}$$

where $S_1^2$ and $S_2^2$ are the two sample variances corresponding to the same feature; $n_1$ and $n_2$ are the two sample capacities corresponding to the same feature, ${\bar{X}}=\frac{\sum _{i=1}^{n} x_{i}}{n}, i=1 \ldots n$.

The nodes are then ranked according to their *p* values, the node with the smallest *p* value is defined as the seed node, and the Pearson correlation coefficient between the remaining nodes and the seed node is calculated. The correlation coefficients are sorted in descending order and the top *p*% of nodes other than the seed nodes are removed. The purpose of this step is to remove the nodes in each cluster that have a high correlation with the seed node. The remaining features in each cluster are used to form a new dataset $D^*$. Then an Improved L1 Regularization method is applied on $D^*$. Considering the random nature of the method: for the dataset with sample number *n*, the experiment repeats *n* feature selections and statistically incorporates the weight of each feature and the number of occurrences of each feature as the final feature selection result. The process is shown in Algorithm 1.
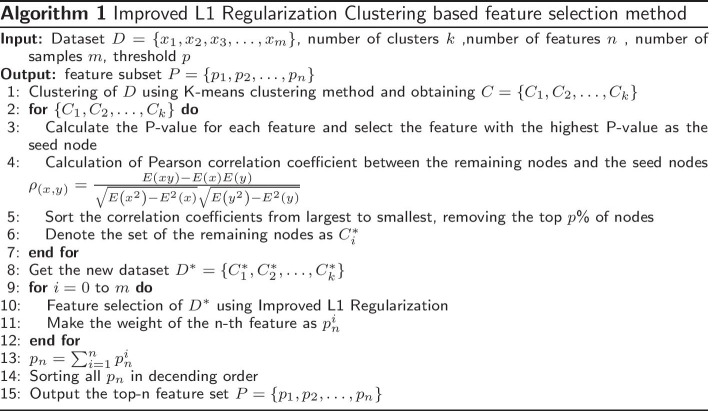

This method can effectively remove redundant features. In an experiment on the CLP data set, we removed the following proteins in one Cluster: P37837, P40197, A0AUP5, B2R701. By reviewing the relevant information on UniProt, we did not find any information indicating that these proteins are related to the clinical diagnosis of CLP, these proteins in this Cluster are removed for the clinical diagnosis redundancy.

## Data Availability

The public data set used in our experiment is from the GEO (Gene Expression Omnibus) database, which can be obtained through the following website: https://www.ncbi.nlm.nih.gov/geo. The patient population data used to support the findings of this study have not been made available because the data are supplied by Hospital under license and so cannot be made freely available. Requests for access to these data should be made to the corresponding author. Our code and public datasets are available at https://github.com/xwdshiwo.

## Abbreviations

ILRC: Improved L1 regularization and clustering
AFP: Alpha fetoprotein
RFE: Recursive feature elimination
GA: Genetic algorithm
KNN: K-nearest neighbor
PSO: Particle swarm optimization
DT: Decision tree
RF: Random forest
LR: Lasso regression
MIN: Mutual information maximization
AGA: Adptive genetic algorithm
SFS: Sequential forward selection
ELM: Exterme learning machine
mRMR: Minimum redundancy maximum relevance
SU: Symmetric uncertainty
HSA: Harmonious search algorithm
CLP: Cleft lip and palate

## References

[1] Wang M, Xu Z, Ding A, Kong Y (2018). Genome-wide identification and expression profiling analysis of the xyloglucan endotransglucosylase/hydrolase gene family in tobacco (*Nicotiana tabacum* l.). Genes.

[2] Luo K, Wang G, Li Q, Tao J. An improved SVM-RFE based on $$F$$-statistic and mPDC for gene selection in cancer classification. IEEE Access. 2019;7:147617–28.

[3] Ayyad SM, Saleh AI, Labib LM. Gene expression cancer classification using modified K-nearest neighbors technique. Biosystems 2019;176:41–51. 10.1016/j.biosystems.2018.12.009 30611843

[4] Saeys Y, Inza I, Larrañaga P (2007). A review of feature selection techniques in bioinformatics. Bioinformatics.

[5] Bolón-Canedo V, Sánchez-Marono N, Alonso-Betanzos A, Benítez JM, Herrera F (2014). A review of microarray datasets and applied feature selection methods. Inf Sci.

[6] Lu H, Chen J, Yan K, Jin Q, Xue Y, Gao Z (2017). A hybrid feature selection algorithm for gene expression data classification. Neurocomputing.

[7] Salem H, Attiya G, El-Fishawy N (2017). Classification of human cancer diseases by gene expression profiles. Appl Soft Comput.

[8] Alshamlan H, Badr G, Alohali Y (2015). mRMR-ABC: a hybrid gene selection algorithm for cancer classification using microarray gene expression profiling. Biomed Res Int.

[9] Jain I, Jain VK, Jain R (2018). Correlation feature selection based improved-binary particle swarm optimization for gene selection and cancer classification. Appl Soft Comput.

[10] Moradi P, Gholampour M (2016). A hybrid particle swarm optimization for feature subset selection by integrating a novel local search strategy. Appl Soft Comput.

[11] Shreem SS, Abdullah S, Nazri MZA (2016). Hybrid feature selection algorithm using symmetrical uncertainty and a harmony search algorithm. Int J Syst Sci.

[12] Djellali H, Guessoum S, Ghoualmi-Zine N, Layachi S. Fast correlation based filter combined with genetic algorithm and particle swarm on feature selection. In: 2017 5th International conference on electrical engineering-Boumerdes (ICEE-B). IEEE; 2017. p. 1–6.

[13] Hoellerer S, Papaxanthos L, Gumpinger AC, Fischer K, Beisel C, Borgwardt K, Benenson Y, Jeschek M. Large-scale DNA-based phenotypic recording and deep learning enable highly accurate sequence-function mapping. bioRxiv (2020). 10.1038/s41467-020-17222-4 PMC7363850 32669542

[14] Liang L, Rasmussen M-LH, Piening B, Shen X, Chen S, Röst H, Snyder JK, Tibshirani R, Skotte L, Lee NC (2020). Metabolic dynamics and prediction of gestational age and time to delivery in pregnant women. Cell.

[15] Chierici M, Bussola N, Marcolini A, Francescatto M, Zandonà A, Trastulla L, Agostinelli C, Jurman G, Furlanello C. Integrative network fusion: a multi-omics approach in molecular profiling. bioRxiv (2020). 10.3389/fonc.2020.01065 PMC7340129 32714870

[16] Norman KC, O’Dwyer DN, Salisbury ML, DiLillo KM, Lama VN, Xia M, Gurczynski SJ, White ES, Flaherty KR, Martinez FJ (2020). Identification of a unique temporal signature in blood and BAL associated with IPF progression. Sci Rep.

[17] Huang L, Wang L, Hu X, Chen S, Tao Y, Su H, Yang J, Xu W, Vedarethinam V, Wu S (2020). Machine learning of serum metabolic patterns encodes early-stage lung adenocarcinoma. Nat Commun.

[18] Han C-L, Sheng Y-C, Wang S-Y, Chen Y-H, Kang J-H (2020). Serum proteome profiles revealed dysregulated proteins and mechanisms associated with fibromyalgia syndrome in women. Sci Rep.

[19] Pd A, Mg B, Lv A (2019). Ensemble feature selection using election methods and ranker clustering. Inf Sci.

[20] Kolde R, Laur S, Adler P, Vilo J (2012). Robust rank aggregation for gene list integration and meta-analysis. Bioinformatics.

[21] Chen Y, Wang X, Lu S, Huang J, Zhang L, Hu W (2020). The diagnostic accuracy of maternal serum alpha-fetoprotein variants (AFP-L2 and AFP-L3) in predicting fetal open neural tube defects and abdominal wall defects. Clin Chim Acta.

[22] Harrison MR, Adzick NS. The fetus as a patient. Surgical considerations. Ann Surg. 1991;213(4):279. 10.1097/00000658-199104000-00002 PMC1358346 2009009

[23] Kavitha K, Prakasan A, Dhrishya P. Score-based feature selection of gene expression data for cancer classification. In: 2020 Fourth international conference on computing methodologies and communication (ICCMC). IEEE; 2020. p. 261–266.

[24] Hsu H-H, Hsieh C-W, Lu M-D (2011). Hybrid feature selection by combining filters and wrappers. Expert Syst Appl.

[25] Chen J, Song A, Zhang W (2011). A novel hybrid gene selection approach based on ReliefF and FCBF. Int J Digit Content Technol Appl.

[26] Zhang Y, Ding C, Li T (2008). Gene selection algorithm by combining ReliefF and MRMR. BMC Genomics.

[27] Kononenko I, Šimec E, Robnik-Šikonja M (1997). Overcoming the myopia of inductive learning algorithms with ReliefF. Appl Intell.

[28] Zhou N, Wang L (2007). A modified t-test feature selection method and its application on the HapMap genotype data. Genomics Proteomics Bioinform.

[29] Liu H, Setiono R. Chi2: feature selection and discretization of numeric attributes. In: Proceedings of 7th IEEE international conference on tools with artificial intelligence. IEEE; 1995. p. 388–391.

[30] Lin C, Miller T, Dligach D, Plenge R, Karlson E, Savova G. Maximal information coefficient for feature selection for clinical document classification. In: ICML workshop on machine learning for clinical data. Edingburgh. 2012.

[31] Raileanu LE, Stoffel K (2004). Theoretical comparison between the gini index and information gain criteria. Ann Math Artif Intell.

[32] Hall M. Smith L. Practical feature subset selection for machine learning. In: Proceedings of the 21st Australasian Computer Science Conference; 1996. vol 98.

[33] Gu Q, Li Z, Han J. Generalized fisher score for feature selection. arXiv preprint arXiv:1202.3725 (2012).

[34] He X, Cai D, Niyogi P. Laplacian score for feature selection. In: Advances in neural information processing systems; 2005. vol 18.

[35] Wang A, An N, Yang J, Chen G, Li L, Alterovitz G (2017). Wrapper-based gene selection with Markov blanket. Comput Biol Med.

[36] Haury A-C, Mordelet F, Vera-Licona P, Vert J-P (2012). TIGRESS: trustful inference of gene regulation using stability selection. BMC Syst Biol.

[37] Yan K, Zhang D (2015). Feature selection and analysis on correlated gas sensor data with recursive feature elimination. Sens Actuators B Chem.

[38] Li X, Xiao N, Claramunt C, Lin H (2011). Initialization strategies to enhancing the performance of genetic algorithms for the p-median problem. Comput Ind Eng.

[39] Kar S, Sharma KD, Maitra M (2015). Gene selection from microarray gene expression data for classification of cancer subgroups employing PSO and adaptive k-nearest neighborhood technique. Expert Syst Appl.

[40] Trelea IC (2003). The particle swarm optimization algorithm: convergence analysis and parameter selection. Inf Process Lett.

[41] Stein G, Chen B, Wu AS, Hua KA. Decision tree classifier for network intrusion detection with GA-based feature selection. In: Proceedings of the 43rd annual Southeast regional conference—volume 2; 2005. p. 136–141.

[42] Chen K-H, Wang K-J, Tsai M-L, Wang K-M, Adrian AM, Cheng W-C, Yang T-S, Teng N-C, Tan K-P, Chang K-S (2014). Gene selection for cancer identification: a decision tree model empowered by particle swarm optimization algorithm. BMC Bioinform.

[43] Fonti V, Belitser E (2017). Feature selection using lasso. VU Amst Res Paper Bus Anal.

[44] Almugren N, Alshamlan H (2019). A survey on hybrid feature selection methods in microarray gene expression data for cancer classification. IEEE Access.

[45] Yan X, Nazmi S, Erol BA, Homaifar A, Gebru B, Tunstel E (2020). An efficient unsupervised feature selection procedure through feature clustering. Pattern Recognit Lett.

[46] Zhu P, Xu Q, Hu Q, Zhang C (2018). Co-regularized unsupervised feature selection. Neurocomputing.

[47] Hasan MM, Basith S, Shamima KM, Lee G, Kurata H. Meta-i6mA: an interspecies predictor for identifying DNA $$N^6$$-methyladenine sites of plant genomes by exploiting informative features in an integrative machine-learning framework. Brief Bioinform. 2020;22:bbaa202. 10.1093/bib/bbaa202 32910169

[48] Mehedi HM, Nalini S, Shaherin B, Gwang L, Watshara S, Balachandran M (2020). HLPpred-Fuse: improved and robust prediction of hemolytic peptide and its activity by fusing multiple feature representation. Bioinformatics.

[49] Mehedi HM, Ashad AM, Watshara S, Deng HW, Balachandran M, Hiroyuki K. NeuroPred-FRL: an interpretable prediction model for identifying neuropeptide using feature representation learning. Brief Bioinform. 2021. 10.1093/bib/bbab167. 10.1093/bib/bbab167 33975333

[50] Zhong L, Gao X, Wang Z (2015). A new kind of improving Somte algorithm based on k-means in imbalanced datasets. Math Pract Theory.

[51] Golub TR, Slonim DK, Tamayo P, Huard C, Lander ES (1999). Molecular classification of cancer: class discovery and class prediction by gene monitoring. Science.

[52] Shipp MA, Ross KN, Tamayo P, Weng AP, Kutok JL, Aguiar RC, Gaasenbeek M, Angelo M, Reich M, Pinkus GS (2002). Diffuse large b-cell lymphoma outcome prediction by gene-expression profiling and supervised machine learning. Nat Med.

[53] Singh D, Febbo PG, Ross K, Jackson DG, Manola J, Ladd C, Tamayo P, Renshaw AA, D’Amico AV, Richie JP (2002). Gene expression correlates of clinical prostate cancer behavior. Cancer Cell.

[54] Chiaretti S, Li X, Gentleman R, Vitale A, Vignetti M, Mandelli F, Ritz J, Foa R (2004). Gene expression profile of adult t-cell acute lymphocytic leukemia identifies distinct subsets of patients with different response to therapy and survival. Blood.

[55] Pomeroy SL, Tamayo P, Gaasenbeek M, Sturla LM, Angelo M, McLaughlin ME, Kim JY, Goumnerova LC, Black PM, Lau C (2002). Prediction of central nervous system embryonal tumour outcome based on gene expression. Nature.

[56] Alizadeh AA, Eisen MB, Davis RE, Ma C, Lossos IS, Rosenwald A, Boldrick JC, Sabet H, Tran T, Yu X (2000). Distinct types of diffuse large b-cell lymphoma identified by gene expression profiling. Nature.

[57] Notterman DA, Alon U, Sierk AJ, Levine AJ (2001). Transcriptional gene expression profiles of colorectal adenoma, adenocarcinoma, and normal tissue examined by oligonucleotide arrays. Cancer Res.

[58] Alon U, Barkai N, Notterman DA, Gish K, Ybarra S, Mack D, Levine AJ (1999). Broad patterns of gene expression revealed by clustering analysis of tumor and normal colon tissues probed by oligonucleotide arrays. Proc Natl Acad Sci.

[59] Golub T.R, Slonim D.K, Tamayo P, Huard C, Gaasenbeek M, Mesirov J.P, Coller H, Loh M.L, Downing J.R, Caligiuri M.A (1999). Molecular classification of cancer: class discovery and class prediction by gene expression monitoring. Science.

[60] Tian E, Zhan F, Walker R, Rasmussen E, Ma Y, Barlogie B, Shaughnessy JD (2003). The role of the Wnt-signaling antagonist DKK1 in the development of osteolytic lesions in multiple myeloma. N Engl J Med.

[61] Wu Y, Grabsch H, Ivanova T, Tan IB, Murray J, Ooi CH, Wright AI, West NP, Hutchins GG, Wu J (2013). Comprehensive genomic meta-analysis identifies intra-tumoural stroma as a predictor of survival in patients with gastric cancer. Gut.

[62] Krug T, Gabriel JP, Taipa R, Fonseca BV, Domingues-Montanari S, Fernandez-Cadenas I, Manso H, Gouveia LO, Sobral J, Albergaria I (2012). Ttc7b emerges as a novel risk factor for ischemic stroke through the convergence of several genome-wide approaches. J Cereb Blood Flow Metab.

[63] For Disease Control C, Prevention, et al. Facts about cleft lip and cleft palate. CDC.gov (http://www.cdc.gov/ncbddd/birthdefects/cleftlip.html). Accessed 14 Feb 2017 (2014).

[64] Reynolds K, Kumari P, Rincon LS, Gu R, Ji Y, Kumar S, Zhou CJ (2019). Wnt signaling in orofacial clefts: crosstalk, pathogenesis and models. Disease Models Mech.

[65] Honein M, Rasmussen S, Reefhuis J, Moore C, Romitti P, Correa A, Watkins M, Lammer E (2004). Maternal smoking, environmental tobacco smoke, and the risk of oral clefts. Am J Epidemiol.

[66] Correa A, Gilboa SM, Besser LM, Botto LD, Moore CA, Hobbs CA, Cleves MA, Riehle-Colarusso TJ, Waller DK, Reece EA (2008). Diabetes mellitus and birth defects. Am J Obstet Gynecol.

[67] Margulis AV, Mitchell AA, Gilboa SM, Werler MM, Mittleman MA, Glynn RJ, Hernandez-Diaz S, Study NBDP (2012). Use of topiramate in pregnancy and risk of oral clefts. Am J Obstet Gynecol.

[68] Werler MM, Ahrens KA, Bosco JL, Mitchell AA, Anderka MT, Gilboa SM, Holmes LB, Study TNBDP (2011). Use of antiepileptic medications in pregnancy in relation to risks of birth defects. Ann Epidemiol.

[69] Worley ML, Patel KG, Kilpatrick LA (2018). Cleft lip and palate. Clin Perinatol.

[70] Reynolds K, Zhang S, Sun B, Garland MA, Ji Y, Zhou CJ (2020). Genetics and signaling mechanisms of orofacial clefts. Birth Defects Res.

[71] Khan MHR, Bhadra A, Howlader T. Stability selection for lasso, ridge and elastic net implemented with AFT models[J]. Stat Appl Genet Molecular Biol. 2019;18(5). 10.1515/sagmb-2017-0001 10.1515/sagmb-2017-0001 31586968

[72] Bradshaw RA (2006). Reporting protein identification data the next generation of guidelines. Mol Cell Proteomics.

[73] Meinshausen N, Bühlmann P (2010). Stability selection. J R Stat Soc Ser B Stat Methodol.

[74] Guo S, Guo D, Chen L, Jiang Q (2017). A l1-regularized feature selection method for local dimension reduction on microarray data. Comput Biol Chem.

[75] Zhou Y, Rong J, Steven H. Exclusive lasso for multitask feature selection. J Mach Learn Res - JMLR. 2010;9:988–995.

[76] Chormunge S, Jena S (2018). Correlation based feature selection with clustering for high dimensional data. J Electr Syst Inf Technol.

[77] Witten DM, Tibshirani R (2010). A framework for feature selection in clustering. J Am Stat Assoc.

[78] Bholowalia P, Kumar A. EBK-Means: A clustering technique based on elbow method and K-Means in WSN. Int J Comput Appl. 2014;105(9):17–24.

